# Cornea nerve fiber quantification and construction of phenotypes in patients with fibromyalgia

**DOI:** 10.1038/srep23573

**Published:** 2016-03-23

**Authors:** Linda Oudejans, Xuan He, Marieke Niesters, Albert Dahan, Michael Brines, Monique van Velzen

**Affiliations:** 1 Department of Anesthesiology, Leiden University Medical Center, Leiden, The Netherlands; 2 Araim Pharmaceuticals Inc., Tarrytown, NY, USA

## Abstract

Cornea confocal microscopy (CCM) is a novel non-invasive method to detect small nerve fiber pathology. CCM generally correlates with outcomes of skin biopsies in patients with small fiber pathology. The aim of this study was to quantify the morphology of small nerve fibers of the cornea of patients with fibromyalgia in terms of density, length and branching and further phenotype these patients using standardized quantitative sensory testing (QST). Small fiber pathology was detected in the cornea of 51% of patients: nerve fiber length was significantly decreased in 44% of patients compared to age- and sex-matched reference values; nerve fiber density and branching were significantly decreased in 10% and 28% of patients. The combination of the CCM parameters and sensory tests for central sensitization, (cold pain threshold, mechanical pain threshold, mechanical pain sensitivity, allodynia and/or windup), yielded four phenotypes of fibromyalgia patients in a subgroup analysis: one group with normal cornea morphology without and with signs of central sensitization, and a group with abnormal cornea morphology parameters without and with signs of central sensitization. In conclusion, half of the tested fibromyalgia population demonstrates signs of small fiber pathology as measured by CCM. The four distinct phenotypes suggest possible differences in disease mechanisms and may require different treatment approaches.

Fibromyalgia is characterized by chronic widespread pain accompanied by a range of symptoms including headache, fatigue, cognitive dysfunction, depression and sleep disturbances^1^,^2^. Diagnosis is based on symptoms that persist for at least three months and that are not explained by any other disease process. Since there is no clear and well-described pathophysiological substrate, fibromyalgia has long been considered a pain state that originates at central sites, *i.e.*, within the central nervous system. Evidence for this hypothesis comes from observations of increased neuronal activity during non-noxious stimulation in brain regions involved in pain processing, and indications of dysfunctional endogenous pain modulatory systems^2^,^3^,^4^. However, recent evidence suggests involvement of the peripheral nervous system in some patients with fibromyalgia. Proof of small fiber involvement comes from studies using skin biopsies, cornea confocal microscopy (CCM) and quantitative sensory testing (QST)^5^,^6^,^7^,^8^,^9^,^10^. For example, Ramirez *et al.*^9^ observed in a small cohort of patients with fibromyalgia a 20% reduction of cornea nerve fiber density compared to control subjects. Additionally, there is proof of abnormal C-fiber nociceptor activity with hyperexcitability in patients with fibromyalgia, very similar to observations in patients with established small fiber neuropathy (SFN)^11^. At this point we would like to mention that with Clauw^12^ and Üçeyler and Sommer^13^ we make a distinction between SFN and small-fiber pathology. As stated by these authors, SFN is reserved for a subgroup of neuropathies in which impairment of small nerve fibers (causing changes in nerve density and autonomic functions) leads to superficial burning pain and abnormal sensations affecting predominantly the feet and hands of the patient. In fibromyalgia we use the term small fiber pathology or small fiber damage as these patients predominantly report deep pain in muscles and tendons and it remains currently unknown what the role is of the small-fiber pathology in the cause of symptoms of fibromyalgia^12^,^13^.

The diagnosis of small fiber pathology is usually based on assessment of neuropathic symptoms, quantitative sensory testing, electromyography and/or skin biopsies, with skin biopsies considered the gold standard for diagnosis. In addition to the invasive nature, intra-observer and intra-patient variability contributes to difficulties when using skin biopsies to diagnose peripheral neuropathy^14^. Cornea confocal microscopy is a relatively novel technique that has been developed to quantify small nerve fibers in the cornea^15^,^16^. CCM examines the densely innervated cornea as a surrogate for the small nerve fiber state, and can serve as a quantitative and qualitative measure of small fiber morphology in a reproducible, non-invasive manner. In most studies, nerve fiber counts in the cornea are generally correlated with skin biopsies, and correlate well with clinical symptoms of small fiber neuropathy especially in patients with patchy neuropathy^16^,^17^,^18^.

To our knowledge, a comprehensive study in fibromyalgia patients including corneal nerve quantification, sensory testing, and questionnaires, is lacking. Combinatory testing aids in the construction of patient phenotypes, identifying possible differences in disease mechanisms that could steer clinical decision-making. The main aim of the current study was to quantify the heterogeneity of the fibromyalgia patient population and assess whether multiple subgroups with distinct phenotypes may be detected based on the morphological state of small fibers and standardized quantitative sensory testing and neuropathic pain questionnaires (PainDetect and small fiber neuropathy screening list, SFNSL). We hypothesized that small fiber pathology, as detected by CCM, is present in a subset of patients with fibromyalgia and that abnormalities in cornea small fiber morphology overlap with abnormalities in QST and questionnaires.

## Results

### Patient demographics and patient-reported symptoms

Patient characteristics of 39 patients that completed the study are given in Table 1. Data from one patient were not included in the analysis due to difficulty in obtaining reliable sensory assessments (QST). Of the remaining 39 patients, 3 were male. Fibromyalgia symptoms were present for 15 years (mean, range 2–37 years). The average number of positive tender points was 14 (range 4–18); 5 patients (13%) had less than 11 tender points. The average widespread pain index was 14 (range 6–18) and the average symptom severity score was 8 (range 4–12). With these ratings, the diagnosis of fibromyalgia was re-confirmed in all patients.

### Cornea Confocal Microscopy

**Figure 1:**
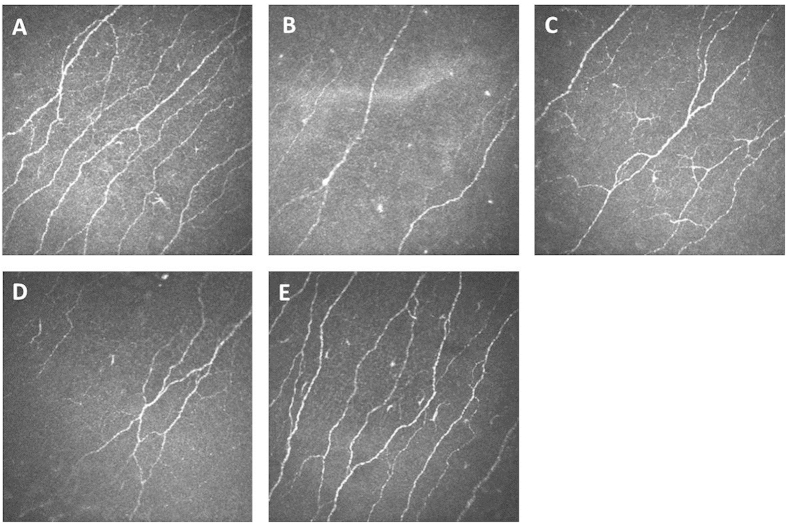
Representative cornea confocal images of fibromyalgia patients compared to healthy volunteers. Confocal microscope images from the cornea nerve plexus. (**A**) 19-year-old healthy female with normal cornea nerve fiber state. (**B**) 21-year-old female patient with significantly decreased cornea nerve fiber state. (**C**) 57-year-old female patient with normal cornea nerve fiber state. (**D**) 58-year-old female patient with significantly decreased cornea nerve fiber state. (**E**) 53-year old male with normal nerve fiber state. Images were acquired with a field of view of 400 × 400 μm.

Figure 1 shows examples of confocal microscope photos of the cornea nerve plexus. Figure 1B shows the cornea plexus of a 21-year-old female fibromyalgia patient with a clear decrease in cornea nerve fiber density (CNFD), cornea nerve branching density (CNBD) and cornea nerve fiber length (CNFL) as compared to age- and sex-matched controls. In comparison, the cornea of a healthy 19-year-old female with normal cornea nerve fiber state is given in Fig. 1A (this photo is derived from a cohort of healthy individuals in our database). Additional images illustrate the corneas of a 57-year-old female patient with a normal cornea nerve fiber state (C), a 58-year female with an abnormal state (reduced CNFL) as compared to age- and sex-matched controls (D)^19^, and a 53-year-old male with a normal cornea nerve fiber density (E). These data demonstrate the effect of disease but also age and sex on the cornea nerve plexus.

**Figure 2:**
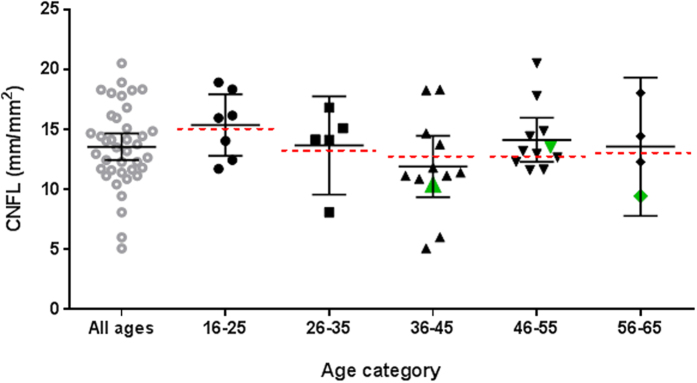
Cornea nerve fiber length (CNFL) individual data (with average and 95% confidence interval) compared to female reference values per age category^19^. The dotted lines indicate the 0.05 percentile normative cutoff values for decreased CNFL. The green data points mark male fibromyalgia patients; only the CNFL of the male in age category 46–55 was not significantly decreased compared to the male reference value (not shown) in the corresponding age category.

Cornea nerve quantification of the left and right eye in each patient was similar (data not shown). Data were therefore averaged per patient. Average values (95% confidence intervals) for CNFD, CNFL and CNBD are given in Table 2. Compared to recently published reference values^19^, abnormalities in cornea nerve fiber morphology were observed in 10–44% of patients. Forty-four percent of patients had CNFL values below the 0.05^th^ percentile of their age and sex reference group. As reference values are given per age group, individual scores are presented per age categories in Fig. 2. Similarly, CNFD and CNBD were below the 0.05^th^ percentile of controls in 10% and 28%, respectively. Abnormalities in cornea morphology were correlated. For example, CNFL values correlated with CNBD (Pearson’s r = 0.81) and CNFD (Pearson’s r = 0.90) (p < 0.01).

### PainDetect questionnaire and Small Fiber Neuropathy Screening List

The average PainDetect score was 19 (range 8–30) points (Table 1). Twenty-two patients (56%) had a score above the neuropathy cutoff of 18 points (out of the possible maximum of 38 points). The SFNSL average score was 32 (range 11–64) points (out of the possible maximum of 84; Table 1). Fifteen patients (38%) scored ≥37 where the presence of small fiber neuropathy becomes highly likely.

### Quantitative Sensory Testing

**Figure 3:**
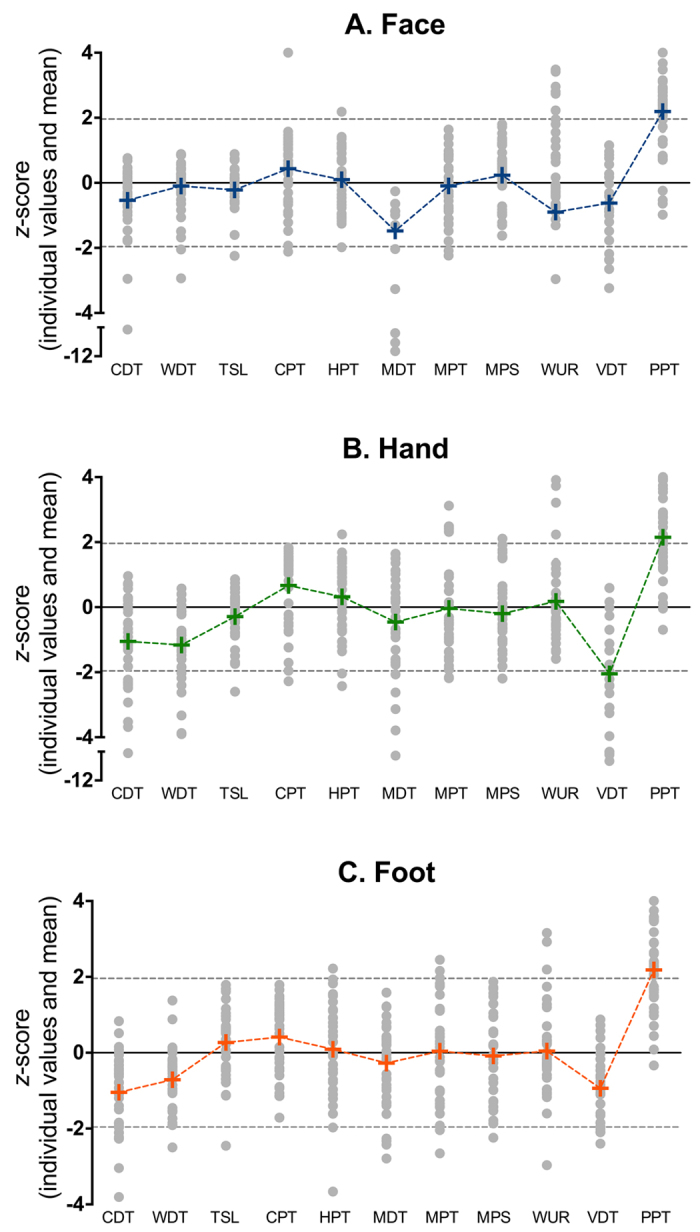
QST profiles of face (**A**), hand (**B**) and foot (**C**) of patients with fibromyalgia. CDT = cold detection threshold; WDT = warm detection threshold; TSL = thermal sensory limen; CPT = cold pain threshold; HPT = heat pain threshold; MDT = mechanical detection threshold; MPT = mechanical pain threshold; MPS = mechanical pain sensitivity; WUR = wind-up ratio; VDT = vibration detection threshold; PPT = pressure pain threshold. The dotted lines indicate ± 1.96*Z above or below which values are considered abnormal. Paradoxical heat sensation and dynamic mechanical allodynia were scored dichotomously and are therefore not included in this figure (see Tables 3 and 4). Each grey dot represents the result observed in one patient. The + signs indicate the mean values.

QST analysis showed that a large number of patients displayed abnormalities with signs of allodynia and hyperalgesia in one or more tested regions (Tables 3 and 4 and Fig. 3). Most important observations include hyperalgesia for mechanical pain (in 21% of patients), wind-up (26%) and pressure pain stimulation (69%) on any of the three test locations. Abnormal sensory detection thresholds were obtained for cold (loss of function), warm (loss of function) and mechanical (loss of function) stimuli in up to 38%, 21% and 23% of patients, respectively. Dynamic mechanical allodynia (gain of function) and paradoxical heat sensations (loss of function) were observed in 13 and 23% of patients, respectively. A loss of vibration sensation was observed in 67% of patients. An overview of the loss and gain of functions in any of the 3 locations is given in Table 3.

### Correlations and subgroup analysis

Pearson’s r showed a strong significant correlation between the SFNSL and PainDetect questionnaires (r = 0.77; p = 0.00). No significant correlations were observed between cornea morphology scores and PainDetect, SFNSL or QST scores. For example, CNFL *vs*. PainDetect: r = −0.11; p = 0.52; CNFL *vs*. SFNSL: r = −0.12; p = 0.47; CNFL *vs*. CDThand: r = 0.21; p = 0.19; CNFL *vs.* MDThand: r = −0.24; p = 0.15; CNFL *vs*. PPThand: r = 0.15; p = 0.37.

Patients with normal and abnormal CNFL values did not differ with respect to QST parameters on face, hand or foot (CDT p = 0.51, WDT p = 0.43, TSL p = 0.99, PHS p = 0.99, CPT p = 0.49, HPT p = 0.99, MDT p = 0.99, MPT p = 0.99, MPS p = 0.99, DMA p = 0.15; WUR p = 0.72; VDT p = 0.99, and PPT p = 0.73), PainDetect (p = 0.74) and SFNSL (p = 0.51) scores. Similarly, these two populations did not differ in the number of tender points (p = 0.08), WPI (p = 0.21), SSS (p = 0.66), age (p = 0.86), BMI (p = 0.69) or years with fibromyalgia symptoms (p = 0.73).

**Figure 4:**
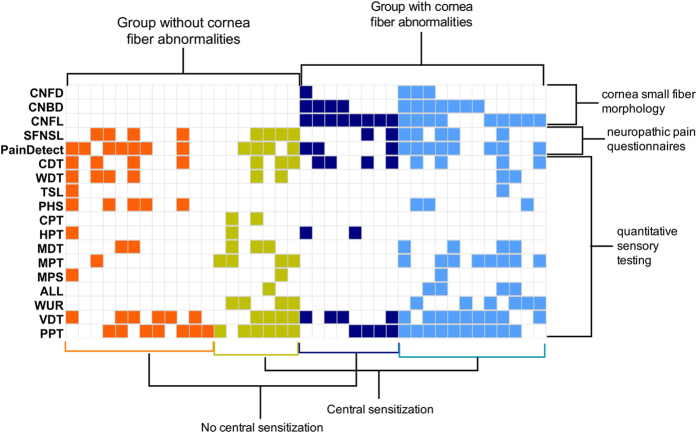
Phenotypes of patients with fibromyalgia based on cornea confocal parameters (CNFD = cornea nerve fiber density; CNBD = cornea nerve branching density; CNFL = cornea nerve fiber length), the small fiber neuropathy screening list (SFNSL), the PainDetect questionnaire and quantitative sensory testing. Columns show normal (white) and abnormal (colored) results per patient. CDT = cold detection threshold; WDT = warm detection threshold; TSL = thermal sensory limen; PHS = paradoxical heat sensation; CPT = cold pain threshold; HPT = heat pain threshold; MDT = mechanical detection threshold; MPT = mechanical pain threshold; MPS = mechanical pain sensitivity; ALL = dynamic mechanical allodynia; WUR = wind-up ratio; VDT = vibration detection threshold; PPT = pressure pain threshold. Colored squares indicate abnormalities in the tests: for cornea confocal microscopy testing values outside the 95% interval of normal reference data, for QST either a gain- or loss-of-function (<−1.96*Z or >1.96*Z) and for the questionnaires values indicative of neuropathic pain.

We defined four subgroups of fibromyalgia patients, based on cornea morphology and signs of central sensitization (as defined by abnormalities in cold pain threshold, mechanical pain threshold, mechanical pain sensitivity, allodynia and/or windup)^20^,^21^. These four subgroups consisted of a group with normal cornea morphology without (n = 12, 31%) and with (n = 7, 18%) signs of central sensitization, and a group with abnormal cornea morphology parameters without (n = 8, 21%) and with (n = 12, 31%) signs of central sensitization (Fig. 4).

## Discussion

The main aim of this study was to assess the involvement of small fiber pathology in patients with fibromyalgia as quantified by CCM and to relate cornea morphology results to patient-reported symptoms and standardized QST. We extended observations by Ramirez *et al.*^9^ who reported that cornea nerve fiber density is abnormal in seventeen patients with fibromyalgia. In our study, CCM analysis revealed at least one significant reduction in one of the small fiber cornea morphology parameters in 51% of fibromyalgia patients when compared to age- and sex-matched reference values. None of the CCM-derived parameter abnormalities were specifically related to age, BMI, questionnaire scores, or QST results.

CCM is a relatively new, non-invasive method to analyze the quantity and quality of small nerve fibers in the cornea. The technique has been validated in several studies involving patients with peripheral neuropathy from various underlying causes, and most studies demonstrate good correlation with intra-epidermal nerve fiber density results from skin biopsies^15^,^17^,^22^,^23^,^24^. CCM has proven to be a sensitive and reproducible measure of peripheral neuropathy and because it is non-invasive, it is an attractive alternative to skin biopsies.

In the past 5 years multiple efforts have been made to establish small fiber pathology in patients with fibromyalgia^5^,^7^,^8^,^10^,^25^, but only one study focused on the cornea^9^. Consistent with our results, in all of these studies subgroups of patients with fibromyalgia were identified that displayed small fiber pathology or (indirect) indications of such pathology. However, as in our study these changes often correlated poorly with symptomatology and neurologic or immunologic measurements. Doppler and colleagues^6^ recently assessed dermal unmyelinated nerve fiber diameter of skin biopsies of the distal and proximal leg and index finger in patients with fibromyalgia and patients with non-diabetic small fiber neuropathy (SFN). They observed that nerve fiber diameter was reduced in patients with fibromyalgia, but not in patients with SFN. The authors concluded that the pathological mechanism underlying small fiber damage might differ between the two disorders, and that patients with fibromyalgia suffer from small fiber *pathology* rather than SFN. This difference in terminology^13^ is a matter of debate and relates to the mechanism of disease; see for example the recent editorial on this topic by Clauw^12^ and letter by Üçeyler and Sommer^13^. Rather than considering small fiber neuropathy as the cause of pain and other symptoms in fibromyalgia, these authors contend that small fiber pathology in fibromyalgia should be treated as an adjunct finding since a cause-effect relationship between the small fiber abnormalities and disease symptomatology has not been established. Our results are in agreement with this latter statement, as we observed no correlation between CCM abnormalities and QST, patient reported symptoms, WPI, SSS and disease duration. Additionally, we observed signs of centrally mediated pain in patients that presented with cornea small fiber pathology, consistent with the idea that central and peripheral pathology coexist in fibromyalgia (Fig. 3). Furthermore, we observed that QST parameters CDT, VDT and PPT were markedly reduced in the majority of patients (Fig. 3), suggesting both small fiber (CDT, PPT) and large fiber (VDT abnormal in 67% of patients) dysfunction. The questionnaire results also indicate that small fiber pathology (SFNSL) or a neuropathic pain component (PainDetect) are highly likely in 38% and 56% of patients. Our results indicate that the fibromyalgia syndrome indeed consists of a heterogeneous group of patients with signs of both central and peripheral small and large nerve fiber pathology. Although the average z-score of the vibration detection test is similar to findings by Klauenberg^26^, the large percentage of detected abnormal vibration detection thresholds is not in agreement with earlier findings, where lower percentages of large fiber abnormalities have been described^13^,^26^,^27^. At this moment we do not have a satisfying explanation, although it may be related to the small range of normal reference values^28^ or differences in the type of recruited patients between our and earlier studies. It is of interest to assess large fiber dysfunction in fibromyalgia patients in further detail by electrophysiological testing.

To better define our patient population, we performed a subgroup analysis to further phenotype the fibromyalgia syndrome based on cornea fiber abnormalities and the presence of central sensitization as suggested by QST parameters cold pain threshold, mechanical pain threshold and mechanical pain sensitivity, allodynia and wind-up (Fig. 4)^20^,^21^,^29^. We identified four subgroups based on a distinction between decreased or normal cornea morphology parameters, and a distinction between signs of central sensitization or the lack thereof. The four acquired subgroups consist of a group with normal cornea morphology without and with signs of central sensitization, and a group with abnormal cornea morphology parameters without and with signs of central sensitization. The detection of these four distinct profiles or phenotypes may be related to the mechanism of disease. For example, the symptoms of patients that do not display peripheral nerve pathology in their CCM data are most probably related to pain arising from the central nervous system, either with or without central sensitization. Patients with cornea nerve fiber pathology may have symptoms of peripheral origin, and in 50% of them signs of mixed (peripheral and central) origin are present.

We cannot exclude that in patients with signs of a peripheral origin of pain, central causes of pain may additionally play a role. One of the criteria in the diagnosis of fibromyalgia is widespread pain: the presence of axial pain, bilateral pain, and upper and lower segment pain^30^. In addition, the same comorbidities found in patients with fibromyalgia, such as fatigue, sleep disturbances, irritable bowel syndrome, cognitive deficits and mood disorders also occur in other chronic pain syndromes and central fatigue syndromes^1^. These symptoms all together suggest a role for a central site of origin of fibromyalgia symptoms, including pain. Indeed, several studies found evidence for central sensitization^31^ and dysfunctional pain inhibition^3^ in neurophysiological and functional MRI studies. Other neuroimaging studies demonstrated elevated levels of excitatory neurotransmitters in the brains of patients with fibromyalgia^32^,^33^ and structural or functional changes in brain regions involved in pain processing, sleep and mood^34^,^35^,^36^. However, it is not known whether the central changes found in these studies are the cause of the pain, or a consequence of continuous nociceptive input, thereby augmenting pain from a peripheral source in those patients with signs of peripheral nerve pathology^37^. Additional studies are required to elucidate this matter, also taking into account psychological state and trait and the genetic background of patients with fibromyalgia, since these factors are well-known to influence the development of fibromyalgia^38^,^39^. Finally, we argue that phenotyping patients with fibromyalgia is not only of importance to understand the mechanism of disease but may also be important in the choice of pain medication. For example, we recently showed that patients with sarcoidosis and small fiber neuropathy benefit from ARA290, an erythropoietin analogue acting at the innate repair receptor, which restores peripheral nerve morphology and neuropathic symptoms^40^. It may well be that this same compound will be exclusively effective in patients with fibromyalgia and small fiber pathology while centrally acting drugs, such as pregabalin, are required when no signs of peripheral nerve fiber pathology are present. Future studies should address these hypotheses.

In conclusion, in a small cohort of fibromyalgia patients we observed signs of small fiber pathology in 51% of patients as measured by cornea confocal microscopy. Further profiling these patients shows that four distinct phenotypes were present: a group with normal cornea morphology with and without signs of central sensitization, and a group with abnormal cornea morphology parameters with and without signs of central sensitization. These phenotypes indicate possible differences in disease mechanisms and additionally may steer the clinician in his or her choice of treatment of this complex, multi-factorial disorder. Since this and other previous studies were relatively small, larger cohorts of patients with fibromyalgia are needed to come to definite conclusions regarding the existence of subgroups in sensory testing and involvement of small-fiber pathology in the mechanism of disease.

## Methods

The protocol was approved by the Ethics Committee of the Leiden University Medical Center (Leiden, the Netherlands), and all study procedures were conducted according to GCP guidelines and adhered to the tenets of the Declaration of Helsinki. The study is registered in the Netherlands Trial Register (NTR3769).

### Patients

Forty patients with fibromyalgia were recruited for the study. Inclusion criteria were: age between 18 and 75 years, fibromyalgia diagnosed by a rheumatologist according to the 1990 or 2010 American College of Rheumatologists criteria^30^,^41^, and willing and able to provide informed consent. Exclusion criteria were: inability to read and understand written text in Dutch, a diagnosis diabetes mellitus, glucose intolerance, sarcoidosis or other diseases that are associated with small-fiber neuropathy, presence of a chronic pain condition other than fibromyalgia, prior eye surgery, use of contact lenses, and pregnancy/lactation. All study participants provided oral and written informed consent prior to study procedures. Fibromyalgia was re-assessed by a trained investigator using the 1990 and 2010 ACR criteria: nine bilateral pressure tender points were tested (18 in total) and the widespread pain index and symptom severity scale score were recorded.

### Cornea Confocal Microscopy

Bilateral CCM was performed on 39 patients, using the Rostock Cornea Module with the Heidelberg Retina Tomograph III (Heidelberg, Germany). Images were acquired and quantified as follows: After topical anesthesia of both eyes, the microscope was placed at the surface of the cornea apex. Confocal images were acquired with a field of view of 400 × 400 μm and automatically quantified using ACCmetrics software (provided by the faculty of Medical and Human Sciences of the University of Manchester, United Kingdom). Cornea nerve fiber length (CNFL), cornea nerve fiber density (CNFD), and cornea nerve branching density (CNBD) were quantified after manual selection (in a blinded fashion, by author MvV) of 5 to 10 representative, high-quality images per eye. Taken the good correlation between semi-automated (CCmetrics) and automated (ACCmetrics) corneal nerve fiber quantification^42^,^43^, the data were compared to semi-automated acquired reference values from Tavakoli *et al.*^19^.

### PainDetect questionnaire and Small Fiber Neuropathy Screening List (SFNSL)

To assess neuropathic pain involvement in daily pain, patients filled out the PainDetect questionnaire, a screening tool to detect neuropathic pain symptoms. This questionnaire assesses pain perception over the last 4 weeks and the current pain score, and patients are asked to localize and qualify their pain (burning, prickling, attacks, etc.). A score of 19 or higher indicates that a neuropathic pain component is likely. The validated Dutch version of the PainDetect was used. To screen for small fiber involvement, patients filled out the small fiber neuropathy screening list (SFNSL) which assesses complaints consistent with small fiber involvement such as indigestion, dry eyes, allodynia, tingling sensations, chest pain and others. The Dutch version validated for sarcoidosis patients was used^44^. A score of 37 or higher indicates that the presence of small fiber involvement is highly likely.

### Quantitative Sensory Testing

QST was performed on the face (buccal surface), hand and foot (both dorsal surface) according to the protocol of the German Research Network on Neuropathic Pain using 13 tests per anatomic location^28^. In short, we tested the following modalities: cold and warm detection and pain thresholds (CDT, WDT, CPT HPT), thermal sensory limen (TSL), paradoxical heat sensation (PHS), mechanical detection and pain thresholds (MDT, MPT), mechanical pain sensitivity (MPS), dynamic mechanical allodynia (DMA), wind-up ratio (WUR), vibration detection threshold (VDT) and pressure pain threshold (PPT). Thermal tests were performed with the Pathway ATS device (Medoc, Ramat Yishai, Israel); mechanical detection and pain thresholds were obtained by using 0.2–588.4 mN von Frey filaments (Touch-Test®, Bioseb, France) and the PinPrick Stimulator set (8–512 mN; MRC-systems, Germany); dynamic mechanical allodynia was examined using brush and cotton-top strokes; pin prick for wind-up testing was performed with the PinPrick Stimulator set (8–512 mN; MRC-systems, Germany); vibration detection threshold was tested with a vibrating tuning fork (Martin Rydell Seiffer, Selles Medical, UK); and finally pressure pain threshold was tested with a handheld pressure algometer (Wagner Instruments, Greenwich, CT). For mechanical pain sensitivity, mechanical allodynia and wind-up, patients were asked to report a pain score based on a numerical rating scale (NRS) ranging from 0, no pain to 10, worst pain imaginable. Pain rating was adapted from a 0–100 point scale to a 0–10 point scale, to facilitate scoring in our Dutch population that is used to use a 10-point scoring system in general. QST results are expressed as transformed *z*-scores, according to published reference values^45^, where values less than –1.96 (loss of function) or greater than 1.96 (gain of function) are considered abnormal. Dynamic mechanical allodynia and paradoxical heat sensations were scored dichotomously.

### Statistical analysis

Statistical testing was performed using GraphPad Prism 6 (GraphPad Software, San Diego, CA) and SPSS statistics 20 (IBM SPSS Statistics, Armonk, NY). For correlation analysis Pearson’s correlation coefficient or Spearman’s rho were used. Subgroups of patients (decreased *vs*. normal CNFL) were compared with the Fisher’s exact test for normal *vs.* abnormal results of: the PainDetect and the SFNSL questionnaire; and all QST parameters. Other comparisons between subgroups were evaluated with the Mann Whitney U test. P values < 0.05 were considered significant. Data are presented as average ±95% confidence interval or (range), unless otherwise indicated.

## Additional Information

**How to cite this article**: Oudejans, L. *et al.* Cornea nerve fiber quantification and construction of phenotypes in patients with fibromyalgia. *Sci. Rep.* **6**, 23573; doi: 10.1038/srep23573 (2016).

## Figures and Tables

**Table 1:** Patient characteristics.

Number of patients (n)	39
Females (%)	36 (92)
Age, years, mean (range)	39.2 (19–58)
Body mass index, kg/m2, mean (range)	25.8 (19.6–38)
Years with fibromyalgia symptoms, mean (range)	15 (2–37)
Years with fibromyalgia diagnosis, mean (range)	6 (1–20)
Number of tender points, mean (range)*	14 (4–18)
Widespread pain index (WPI), mean (range)#	14 (6–18)
Symptom severity scale score (SSS), mean (range)#	8 (4–12)
PainDetect questionnaire score, mean (range)$	19 (8–30)
SFNSL total score, mean (range)§	32 (11–64)
SFNSL pain subscore	18 (7–32)
SFNSL autonomic dysfunction subscore	14 (3–33)

^*^A score of 11 points is indicative of fibromyalgia.

^#^A combination of WPI ≥ 7 and SSS ≥ 5 or WPI 3–6 and SS ≥ 9 is indicative of fibromyalgia.

^$^A score of 19 or higher is indicative of a neuropathic component of pain.

^§^A score of ≥37 indicates that the presence of small fiber neuropathy is highly likely. SFNSL: small fiber neuropathy screening list.

**Table 2:** Quantification of cornea nerve fibers in fibromyalgia patients.

Cornea confocal microscopy parameter	Average (95% CI)	Range (min-max)	Significantly decreased, n (%)*
Cornea nerve fiber density (n/mm^2^)	23.3 (21.3–25.3)	10.6–36.9	4 (10)
Cornea nerve branching density (n/mm^2^)	30.5 (26.7–34.3)	4.0–69.0	11 (28)
Cornea nerve fiber length (mm/mm^2^)	13.7 (12.7–14.7)	6.0–20.5	17 (44)

CI: confidence interval. *Relative to reference values^19^.

**Table 3:** Quantitative sensory testing: Number and percentage of fibromyalgia patients with abnormal values on at least one of three test locations: the cheekbone and the dorsal surface of hand and foot.

QST parameter	Average Z-score (95% CI)	Range Z-score (min-max)	Loss of function, n (%)	Gain of function, n (%)
Cold detection threshold	−0.86 (−1.23/−0.48)	−4.69/0.96	15 (38)	–
Warm detection threshold	−0.64 (−0.94/−0.34)	−3.91/1.38	8 (21)	–
Thermal sensory limen	−0.08 (−0.33/0.17)	−2.60/1.79	2 (5)	–
Paradoxical heat sensations	–	–	9 (23)	–
Cold pain threshold	0.50 (0.05/0.95)	−2.28/1.83	1 (3)	1 (3)
Heat pain threshold	0.19 (−0.17/0.55)	−3.66/2.24	2 (5)	2 (5)
Mechanical detection threshold	−0.72 (−1.26/−0.18)	−10.61/1.64	9 (23)	–
Mechanical pain threshold	0.08 (−0.36/0.51)	−2.65/3.12	5 (13)	8 (21)
Mechanical pain sensitivity	−0.01 (−0.39/0.38)	−2.24/2.11	2 (5)	1 (3)
Vibration detection threshold	−1.17 (−1.58/−0.77)	−6.68/1.15	26 (67)	–
Pressure pain threshold	2.68 (2.18/3.18)	−0.98/6.49	–	27 (69)
Temporal summation (wind-up)	−0.08 (−0.10/0.74)	−1.48/3.91	–	10 (26)
Dynamic mechanical allodynia	–	–	–	5 (13)

Percentage of patients with decreased detection thresholds and increased pain thresholds expressed in Z-scores compared to age and sex matched reference values^45^ were calculated from 39 patients. For paradoxical heat sensations and dynamic mechanical allodynia, z-transformations do not yield realistic comparable numbers. Instead, the prevalence of these variables was recorded in a dichotomous fashion.

**Table 4:** Prevalence of paradoxical heat sensations and dynamic mechanical allodynia on face, hand and foot in fibromyalgia patients.

	Paradoxical heat sensations n (%)	Dynamic mechanical allodynia n (%)
face	0 (0)	3 (8)
hand	1 (3)	3 (8)
foot	8 (21)	5 (13)

## References

[b1] Clauw D. J. Fibromyalgia: a clinical review. Jama 311, 1547–1555, doi: 10.1001/jama.2014.3266 (2014). 24737367

[b2] Schmidt-Wilcke T. & Clauw D. J. Fibromyalgia: from pathophysiology to therapy. Nature reviews. Rheumatology 7, 518–527, doi: 10.1038/nrrheum.2011.98 (2011). 21769128

[b3] Jensen K. B. *et al.* Evidence of dysfunctional pain inhibition in Fibromyalgia reflected in rACC during provoked pain. Pain 144, 95–100, doi: 10.1016/j.pain.2009.03.018 (2009). 19410366

[b4] Lautenbacher S. & Rollman G. B. Possible deficiencies of pain modulation in fibromyalgia. The Clinical journal of pain 13, 189–196 (1997). 9303250 10.1097/00002508-199709000-00003

[b5] Caro X. J. & Winter E. F. Evidence of abnormal epidermal nerve fiber density in fibromyalgia: clinical and immunologic implications. Arthritis Rheumatol 66, 1945–1954, doi: 10.1002/art.38662 (2014). 24719395

[b6] Doppler K., Rittner H. L., Deckart M. & Sommer C. Reduced dermal nerve fiber diameter in skin biopsies of patients with fibromyalgia. Pain 156, 2319–2325, doi: 10.1097/j.pain.0000000000000285 (2015). 26164586

[b7] Giannoccaro M. P., Donadio V., Incensi A., Avoni P. & Liguori R. Small nerve fiber involvement in patients referred for fibromyalgia. Muscle & nerve 49, 757–759, doi: 10.1002/mus.24156 (2014). 24469976

[b8] Oaklander A. L., Herzog Z. D., Downs H. M. & Klein M. M. Objective evidence that small-fiber polyneuropathy underlies some illnesses currently labeled as fibromyalgia. Pain 154, 2310–2316, doi: 10.1016/j.pain.2013.06.001 (2013). 23748113 PMC3845002

[b9] Ramirez M. *et al.* Small fiber neuropathy in women with fibromyalgia. An *in vivo* assessment using corneal confocal bio-microscopy. Seminars in arthritis and rheumatism 45, 214–219, doi: 10.1016/j.semarthrit.2015.03.003 (2015). 26094164

[b10] Uceyler N. *et al.* Small fibre pathology in patients with fibromyalgia syndrome. Brain : a journal of neurology 136, 1857–1867, doi: 10.1093/brain/awt053 (2013). 23474848

[b11] Serra J. *et al.* Hyperexcitable C nociceptors in fibromyalgia. Annals of neurology 75, 196–208, doi: 10.1002/ana.24065 (2014). 24243538

[b12] Clauw D. J. What is the meaning of “small fiber neuropathy” in fibromyalgia? Pain 156, 2115–2116, doi: 10.1097/j.pain.0000000000000311 (2015). 26307862

[b13] Uceyler N. & Sommer C. Objective evidence that small-fiber polyneuropathy underlies some illnesses currently labeled as fibromyalgia. Pain 154, 2569, doi: 10.1016/j.pain.2013.06.037 (2013). 23811315

[b14] Wopking S. *et al.* Significant difference between three observers in the assessment of intraepidermal nerve fiber density in skin biopsy. BMC Neurol 9, 13, doi: 10.1186/1471-2377-9-13 (2009). 19335896 PMC2672925

[b15] Petropoulos I. N. *et al.* Corneal nerve loss detected with corneal confocal microscopy is symmetrical and related to the severity of diabetic polyneuropathy. Diabetes Care 36, 3646–3651, doi: 10.2337/dc13-0193 (2013). 23877983 PMC3816900

[b16] Tavakoli M. *et al.* Corneal confocal microscopy: a novel noninvasive test to diagnose and stratify the severity of human diabetic neuropathy. Diabetes Care 33, 1792–1797, doi: 10.2337/dc10-0253 (2010). 20435796 PMC2909064

[b17] Brines M. *et al.* Corneal nerve quantification predicts the severity of symptoms in sarcoidosis patients with painful neuropathy. Technology 1, 1–7 (2013).24999487

[b18] Ziegler D. *et al.* Early detection of nerve fiber loss by corneal confocal microscopy and skin biopsy in recently diagnosed type 2 diabetes. Diabetes 63, 2454–2463, doi: 10.2337/db13-1819 (2014). 24574045

[b19] Tavakoli M. *et al.* Normative values for corneal nerve morphology assessed using corneal confocal microscopy: a multinational normative data set. Diabetes Care 38, 838–843, doi: 10.2337/dc14-2311 (2015). 25633665 PMC4407754

[b20] Arendt-Nielsen L. & Yarnitsky D. Experimental and clinical applications of quantitative sensory testing applied to skin, muscles and viscera. The journal of pain: official journal of the American Pain Society 10, 556–572, doi: 10.1016/j.jpain.2009.02.002 (2009). 19380256

[b21] Woolf C. J. Central sensitization: implications for the diagnosis and treatment of pain. Pain 152, S2–15, doi: 10.1016/j.pain.2010.09.030 (2011). 20961685 PMC3268359

[b22] Malik R. A. *et al.* Corneal confocal microscopy: a non-invasive surrogate of nerve fibre damage and repair in diabetic patients. Diabetologia 46, 683–688, doi: 10.1007/s00125-003-1086-8 (2003). 12739016

[b23] Tavakoli M. & Malik R. A. Corneal confocal microscopy: a novel non-invasive technique to quantify small fibre pathology in peripheral neuropathies. J Vis Exp, doi: 10.3791/2194 (2011). PMC3182640 21248693

[b24] Tavakoli M. *et al.* Corneal confocal microscopy: a novel noninvasive means to diagnose neuropathy in patients with Fabry disease. Muscle & nerve 40, 976–984, doi: 10.1002/mus.21383 (2009). 19902546

[b25] Kosmidis M. L. *et al.* Reduction of Intraepidermal Nerve Fiber Density (IENFD) in the skin biopsies of patients with fibromyalgia: a controlled study. J Neurol Sci 347, 143–147, doi: 10.1016/j.jns.2014.09.035 (2014). 25304055

[b26] Klauenberg S. *et al.* Depression and changed pain perception: hints for a central disinhibition mechanism. Pain 140, 332–343, doi: 10.1016/j.pain.2008.09.003 (2008). 18926637

[b27] Blumenstiel K. *et al.* Quantitative sensory testing profiles in chronic back pain are distinct from those in fibromyalgia. The Clinical journal of pain 27, 682–690, doi: 10.1097/AJP.0b013e3182177654 (2011). 21487289

[b28] Rolke R. *et al.* Quantitative sensory testing in the German Research Network on Neuropathic Pain (DFNS): standardized protocol and reference values. Pain 123, 231–243, doi: 10.1016/j.pain.2006.01.041 (2006). 16697110

[b29] Lang P. M. *et al.* Sensory neuropathy and signs of central sensitization in patients with peripheral arterial disease. Pain 124, 190–200, doi: 10.1016/j.pain.2006.04.011 (2006). 16716518

[b30] Wolfe F. *et al.* The American College of Rheumatology preliminary diagnostic criteria for fibromyalgia and measurement of symptom severity. Arthritis care & research 62, 600–610, doi: 10.1002/acr.20140 (2010). 20461783

[b31] Desmeules J. A. *et al.* Neurophysiologic evidence for a central sensitization in patients with fibromyalgia. Arthritis and rheumatism 48, 1420–1429, doi: 10.1002/art.10893 (2003). 12746916

[b32] Fayed N. *et al.* Localized 1H-NMR spectroscopy in patients with fibromyalgia: a controlled study of changes in cerebral glutamate/glutamine, inositol, choline, and N-acetylaspartate. Arthritis research & therapy 12, R134, doi: 10.1186/ar3072 (2010). 20609227 PMC2945024

[b33] Harris R. E. *et al.* Elevated insular glutamate in fibromyalgia is associated with experimental pain. Arthritis and rheumatism 60, 3146–3152, doi: 10.1002/art.24849 (2009). 19790053 PMC2827610

[b34] Gracely R. H. & Ambrose K. R. Neuroimaging of fibromyalgia. Best practice & research. Clinical rheumatology 25, 271–284, doi: 10.1016/j.berh.2011.02.003 (2011). 22094201

[b35] Jensen K. B. *et al.* Patients with fibromyalgia display less functional connectivity in the brain’s pain inhibitory network. Molecular pain 8, 32, doi: 10.1186/1744-8069-8-32 (2012). 22537768 PMC3404927

[b36] Napadow V. & Harris R. E. What has functional connectivity and chemical neuroimaging in fibromyalgia taught us about the mechanisms and management of ‘centralized’ pain? Arthritis research & therapy 16, 425 (2014). 25606591 10.1186/s13075-014-0425-0 PMC4289059

[b37] Clauw D. J. Diagnosing and treating chronic musculoskeletal pain based on the underlying mechanism(s). Best Pract Res Cl Rh 29, 6–19, doi: 10.1016/j.berh.2015.04.024 (2015). 26266995

[b38] Ablin J. N. & Buskila D. Update on the genetics of the fibromyalgia syndrome. Best practice & research. Clinical rheumatology 29, 20–28, doi: 10.1016/j.berh.2015.04.018 (2015). 26266996

[b39] Desmeules J. *et al.* Central pain sensitization, COMT Val158Met polymorphism, and emotional factors in fibromyalgia. The journal of pain : official journal of the American Pain Society 15, 129–135, doi: 10.1016/j.jpain.2013.10.004 (2014). 24342707

[b40] Heij L. *et al.* Safety and efficacy of ARA 290 in sarcoidosis patients with symptoms of small fiber neuropathy: a randomized, double-blind pilot study. Mol Med 18, 1430–1436, doi: 10.2119/molmed.2012.00332 (2012). 23168581 PMC3563705

[b41] Wolfe F. *et al.* The American College of Rheumatology 1990 Criteria for the Classification of Fibromyalgia. Report of the Multicenter Criteria Committee. Arthritis Rheumatol 33, 160–172 (1990). 10.1002/art.1780330203 2306288

[b42] Chen X. *et al.* Small Nerve Fiber Quantification in the Diagnosis of Diabetic Sensorimotor Polyneuropathy: Comparing Corneal Confocal Microscopy With Intraepidermal Nerve Fiber Density. Diabetes Care, doi: 10.2337/dc14-2422 (2015). PMC4439535 25795415

[b43] Petropoulos I. N. *et al.* Rapid automated diagnosis of diabetic peripheral neuropathy with *in vivo* corneal confocal microscopy. Investigative ophthalmology & visual science 55, 2071–2078, doi: 10.1167/iovs.13-13787 (2014). 24569580 PMC3979234

[b44] Hoitsma E., De Vries J. & Drent M. The small fiber neuropathy screening list: Construction and cross-validation in sarcoidosis. Respiratory medicine 105, 95–100, doi: 10.1016/j.rmed.2010.09.014 (2011). 20889323

[b45] Magerl W. *et al.* Reference data for quantitative sensory testing (QST): refined stratification for age and a novel method for statistical comparison of group data. Pain 151, 598–605, doi: 10.1016/j.pain.2010.07.026 (2010). 20965658

